# Larger Food Portion Sizes Are Associated with Both Positive and Negative Markers of Dietary Quality in Irish Adults

**DOI:** 10.3390/nu10121929

**Published:** 2018-12-05

**Authors:** Jacqueline Lyons, Janette Walton, Albert Flynn

**Affiliations:** School of Food and Nutritional Sciences, University College Cork, T12 K8AF Cork, Ireland; janette.walton@cit.ie (J.W.); a.flynn@ucc.ie (A.F.)

**Keywords:** portion sizes, dietary quality, energy density, adults

## Abstract

Reduction in portion size, particularly for energy-dense foods, is increasingly addressed in healthy eating guidelines in a bid to tackle the obesity epidemic. The effect of portion size on other aspects of dietary quality, such as nutrient intakes, is less studied. The aim of the current work was to investigate associations between food portion sizes and key indicators of dietary quality, namely energy-adjusted intakes of saturated fat, dietary fibre, sodium, calcium, iron, folate and vitamin D, and dietary energy density (DED), in Irish adults on the days the foods were consumed. Data from the Irish National Adult Nutrition Survey (2008–2010) (*n* = 1274, 18–64 years, 4-day semi-weighed record) were used for the analysis. DED was lower on the days larger portions of boiled potatoes, fruit, vegetables and baked beans were consumed, and higher on the days larger portions of white bread, ready-to-eat breakfast cereals (RTEBCs), frying meats, cheese, butter, biscuits, chocolate and sugar-sweetened beverages were consumed. Micronutrient intakes were higher on the days larger portions of brown bread, RTEBCs, vegetables and low-fat spreads were consumed, and lower on the days larger portions of white bread, butter, biscuits, chocolate, sugar-sweetened beverages and beer/cider were consumed, with the exception of folate. The study identifies foods for which larger portion sizes may be associated with positive dietary attributes, as well as the opposite. It provides an important evidence base from which more specific dietary guidance on food portion sizes might be developed for Irish adults.

## 1. Introduction

Research into food portion sizes in adults has traditionally focused on the effects of varying portion sizes on food and energy intake in controlled feeding studies. For both men and women of varying ages and body weights, experimental studies have consistently shown that increasing food portion sizes increases voluntary energy intake. Increased energy intakes occur when American adults are served larger portions of a range of foods, including caloric beverages [1], baked pasta dishes [2], fresh or stale popcorn [3], cookies, yoghurt, garlic bread and chicken stir-fry meals [4]. The association between larger portion sizes and increased energy intake has been shown to be true for foods served as a unit (e.g., a deli-type sandwich) [5], for pre-packaged snack foods (e.g., potato chips) [6] and for foods of amorphous shape (e.g., macaroni and cheese) [7]. Fruit and vegetables may be the only notable exceptions to this trend, with no significant increase in energy intake noted when larger portions of these foods are provided to adults [8]. This is of interest, as it suggests that the relationship between portion size and energy intake may vary according to the food in question. Broadening the portion size question further, we might ask whether aspects of dietary quality other than energy intake will vary according to the portion size of food consumed.

Relationships between food portion sizes and indicators of dietary quality (i.e., dietary energy density (DED), nutrient intakes) have been described for children and adolescents [9]; however, no such work has been published for adults. Public health campaigns addressing the issue of food portion size typically focus on a reduction in the portion size of energy-dense foods to reduce energy intake and help tackle the obesity epidemic. The effect of reducing food portion size on dietary quality, however, is not usually addressed. It is important to investigate this in order to identify foods for which larger portion sizes may be associated with positive dietary attributes, as well as the opposite.

Intakes of several nutrients have been highlighted as being of public health concern for Irish adults in recent years. Significant proportions of Irish adults fall short of the recommended intakes of dietary fibre [10] and vitamin D [11], whilst inadequate intakes of iron, calcium and folate have been reported in Irish women [12]. Intakes of saturated fat as a percentage of total energy (%TE) have been shown to exceed recommendations in Irish adults [13]. An inverse relationship between DED and dietary quality has been reported in this group [14], as have sodium levels in excess of population-average target levels [15].

The aim of the current work was to investigate associations between food portion sizes and key indicators of dietary quality (i.e., energy-adjusted intakes of saturated fat, dietary fibre, sodium, calcium, iron, folate and vitamin D, and DED) in Irish adults aged 18–64 years on the days the foods were consumed.

## 2. Methods

### 2.1. Survey Methodology

The analyses were conducted using data from the Irish National Adult Nutrition Survey (NANS) (2008–2010), which was carried out as part of a series of national food consumption surveys by the Irish Universities Nutrition Alliance (IUNA), a formal association of the nutrition units at University College Cork, University College Dublin, the University of Ulster and Trinity College Dublin. The NANS used a 4-day semi-weighed food record to collect food intake data from adults aged 18 to 90 years (*n* = 1500); the current study includes data from adults aged 18 to 64 years only (*n* = 1274). Adults aged 65 years and upwards should be considered separately in portion size analyses given their decreased estimated average requirements for energy [16], and the smaller food portion weights typically consumed by this group [17]. Participants received training in how to complete the 4-day food record prior to commencing the study. The food record was reviewed in detail by the fieldworker on one occasion during the study period, as well as immediately afterwards, to check for omissions and clarify weights. Participants were required to include at least one weekend day in the consecutive four-day study period to account for possible variations in dietary behaviour over the weekend, such as increased alcohol consumption. Food records were kept during all months of the year to account for seasonal influences on diet. ‘Semi-weighed’ means participants were asked to record weights for as many foods as possible (by using the digital scales provided or by recording manufacturer’s weights on packets), with researchers assigning weights to remaining food items using a variety of quantification tools. Fifty-seven per cent of food items consumed were either directly weighed on the digital scales or assigned a weight using the manufacturer’s information (e.g., one can of soda = 330 mL), representing the most reliable quantification methods available. The remaining food items were assigned weights using, either a photographic food atlas [18], portion-size reference book [19] or household measures. Dietary intake data were converted to nutrient intake data using WISP^© ^(Tinuviel Software, Anglesey, UK), which contains data from *McCance and Widdowson’s The Composition of Foods*, sixth [20] and fifth [21] editions plus all nine supplemental volumes [22,23,24,25,26,27,28,29,30], updated with data from *The Irish Food Composition Database* [31]. The NANS was conducted according to the guidelines laid down in the Declaration of Helsinki. All procedures involving participants were approved by the relevant local ethics committees, and all participants provided written informed consent. Further detail on the survey methodology is available at www.iuna.net [32].

### 2.2. Definition of Food Portion Size

For the current work, food portion size was defined as the weight of food consumed per eating occasion (weight served minus leftovers), and was estimated once for each participant for each day a food was consumed. Where a particular food was consumed on more than one occasion on one day, the largest portion size was used in the analyses, since a larger portion weight is more likely to have an influence on dietary quality on that day. A specific methodology was applied in defining portion weights of ‘beer/cider’. Because, in many cases, such beverages were consumed at various time points throughout an evening and were recorded in the food consumption dataset as ‘one pint of lager consumed at 18.00, 19.30, 21.00’, the portion size was considered to be the amount of the beverage consumed in one day, rather than at one eating occasion. It was thought that this aggregation would allow for a more accurate relationship with dietary quality to be observed, as were it not applied, the portion sizes would merely represent the units in which these beverages are typically consumed (e.g., 500 mL bottle, pint).

### 2.3. Selection of Foods and Dietary Quality Indicators for Analysis

All foods and beverages consumed in the survey were assigned to one of 68 different food groups previously defined by the IUNA research group. Within each food group, foods were ranked by frequency of consumption (i.e., number of eating occasions) to determine key items for analysis. Foods that were very infrequently consumed were excluded from the analysis (e.g., scotch eggs, consumed on one eating occasion only). Similar foods within a food group were aggregated where appropriate (e.g., ‘jellies’, ‘boiled sweets’, ‘chew sweets’, etc. were combined to create the ‘sugary sweets’ group). An extensive selection of foods from all of the major food categories (i.e., starchy foods, fruit and vegetables, milk and dairy foods, meat and other protein foods, foods high in fat or sugar) was included for analysis. ‘Ethnic dishes’ refers to restaurant-prepared dishes like curries, satay or sweet and sour dishes, excluding any starchy component like rice or naan bread. ‘Frying meats’ refers to bacon, sausage and pudding meat products. The dietary quality indicators selected for analysis were energy-adjusted intakes of saturated fat, dietary fibre, Na, Ca, Fe, folate and vitamin D, and DED. These were selected on the basis of their public health relevance to Irish adults, as described previously using data from the same cohort of Irish adults [10,11,12,13,14,15].

### 2.4. Measurement of Dietary Energy Density (DED)

DED is defined as the amount of available dietary energy per unit weight of food or beverage, and is expressed in kJ/g. In the current study, DED was calculated to include all foods and exclude all beverages, as recommended in the most recent systematic review on methods of calculation of DED [33].

### 2.5. Dietary Reference Values (DRVs) Used

Dietary reference values (DRVs) vary for certain nutrients according to whether the population in question consumes alcohol or not [34]. Because 89% of those included in the current study reported that they were alcohol consumers (questionnaire data) and 62% consumed alcohol during the recording period [32], saturated fat intakes were examined using the set of DRVs devised for alcohol consumers, i.e., they were calculated as %TE rather than food energy. Using this set of DRVs assumes an average population intake of 5% energy from alcohol; a figure based on UK intakes [34]. 

### 2.6. Statistical Analyses

Statistical analyses were conducted using the statistical package SPSS^©^ version 15.0 (Chicago, IL, USA) for Windows. Participants defined as energy under-reporters (30%) using the method of Goldberg et al. (1991) were not excluded from the analysis [35]. Some provision was made for the possible effects of under-reporting by reporting energy-adjusted nutrients as outcomes. In addition, it has been reported that the inclusion of energy under-reporters makes little difference to the estimation of typical portion weights in Irish adults, suggesting that in this group, energy under-reporting occurs more typically as the ‘omission’ of food items than the ‘underestimation’ of food portion weights [36].

For each food item examined, the portion size data were split by tertile to create relatively ‘small’ (T1), ‘medium’ (T2) and ‘large’ (T3) portion size tertiles. Each food included for analysis was first examined to determine whether there were significant differences in the portion weights consumed by men and women, or by different age groups (18–35 years, 36–50 years, 51–64 years). Where significant differences occurred, the data were stratified (arranged in layers) so that the resulting three portion size groups represented relatively small, medium and large portions for that food, with each group (e.g., ‘small’ (T1) portions of pizza) containing a mix of men and women from different age groups, all of whom consumed similar portions of the food relative to their peers of the same age group and gender.

Mean values for each of the dietary quality indicators were then compared across portion size tertiles (T1 vs. T2 vs. T3) for the days on which portions of a particular food were consumed. A one-way between-groups analysis of variance (ANOVA) with a Tukey post-hoc comparison test was used to test for significant differences in means across tertiles where dietary quality indicators were normally distributed (*p* < 0.05). Kruskal–Wallis tests with Mann–Whitney U Test comparisons were used where the data were not normally distributed (*p* < 0.05). Normality was assessed using a Kolmogorov–Smirnov test. Following analysis, the Holm adjustment was manually applied to significant results to reduce the probability of Type 1 errors occurring. This adjustment involved ordering test results from the smallest to the largest *p*-value, and testing the smallest probability with a Bonferroni correction (i.e., multiplying p-value by the total number of tests performed). If the first test was significant, then the second smallest probability was tested (i.e., *p*-value multiplied by total number of tests performed minus one), and so on. The procedure ended when the first non-significant test had been obtained, or all tests had been performed.

## 3. Results

Table 1 presents baseline demographic and lifestyle data on the study participants.

Table 2 describes the number of eating occasions of each of the food items included for analysis, and the median portion weights consumed in each of the ‘small’ (T1), ‘medium’ (T2) and ‘large’ (T3) portion size tertiles.

Table 3 describes associations between dietary quality indicators and portion sizes of a range of foods on the days they were consumed in Irish adults, as summarized:

Intakes of saturated fat were higher on the days larger portions of frying meat, whole milk, cheese, butter and chocolate were consumed, and lower on the days larger portions of fruit, baked beans, sugar-sweetened beverages and beer/cider were consumed.

Intakes of dietary fibre were higher on the days larger portions of brown bread, ready-to-eat breakfast cereals (RTEBCs), fruit, vegetables and baked beans were consumed, and lower on the days larger portions of white bread, frying meats, cheese, chocolate, sugary sweets, sugar-sweetened beverages and beer/cider were consumed.

DED was higher on the days larger portions of white bread, RTEBCs, frying meats, cheese, butter, biscuits, chocolate and sugar-sweetened beverages were consumed, and lower on the days larger portions of boiled potatoes, fruit, vegetables and baked beans were consumed.

Intakes of sodium were higher on the days larger portions of white bread, brown bread, baked beans, frying meats and ethnic dishes were consumed, and lower on the days larger portions of chocolate and beer/cider were consumed.

Intakes of calcium were higher on the days larger portions of white bread, brown bread, whole milk, reduced fat milks and cheese were consumed, and lower on the days larger portions of fish, biscuits, crisps and beer/cider were consumed.

Intakes of iron were higher on the days larger portions of brown bread, RTEBCs and vegetables were consumed, and lower on the days larger portions of white bread, cheese, butter, chocolate, sugar-sweetened beverages and beer/cider were consumed.

Intakes of total folate were higher on the days larger portions of RTEBCs, vegetables, low-fat spreads and beer/cider were consumed, and lower on the days larger portions of white bread, pizza, frying meats, butter, biscuits and sugar-sweetened beverages were consumed.

Intakes of vitamin D were higher on the days larger portions of low-fat spreads were consumed, and lower on the days larger portions of white bread, chocolate, sugar-sweetened beverages and beer/cider were consumed.

## 4. Discussion

The current study describes, for the first time, associations between food portion sizes and markers of dietary quality in Irish adults. Research into food portion sizes has traditionally focused on portion size in relation to energy intakes in both experimental and observational work. The current work takes a different approach by examining the relationship between portion size of a range of foods and other markers of dietary quality (e.g., DED, energy-adjusted nutrient intakes). We know of no other study to have examined these associations in adults, and believe our findings to be potentially useful in the development of dietary guidance around food portion sizes.

A majority of Irish adults are known to exceed the UK Department of Health population goal for saturated fat (≤11% TE) [34], with reported mean daily intakes of 13.3% TE [13]. The current work showed an increase in saturated fat intakes with increasing portions of whole milk and butter, but not reduced fat milk or low-fat spreads. These findings are in keeping with current dietary guidelines in Ireland, which recommend choosing reduced fat milk and spreads in place of the full fat varieties [37]. Saturated fat intakes were seen to drop dramatically with increasing portion size of beer/cider, with mean intakes as low as 8.6% TE on the days larger portions were consumed. However, this is likely to represent a proportional increase in the energy provided by alcohol on those days, rather than any conscious efforts to maintain low saturated fat intakes by this group.

A high prevalence of inadequate dietary fibre intakes has been reported in the Irish population [10], with over 80% of adults not meeting the European Food Safety Authority (EFSA) recommendation of 25 g/day [38]. The current study showed very high dietary fibre intakes in adults on the days larger portions of baked beans were consumed (38 g/10 MJ compared to a mean value of 23 g/10 MJ for the whole group). Dietary fibre intakes increased significantly with increasing portion size of brown bread, but decreased with increasing portion size of white bread; again, supporting current dietary guidelines in Ireland which recommend choosing ‘mostly wholegrain’ breads for digestive health [37]. This finding highlights the impact a switch from white to brown bread could make to dietary fibre intakes at an individual level.

Mean dietary salt intakes in Irish adults aged 18 to 64 years are in excess of the population-average target of 6 g/day for adults [39]. Larger portions of frying meats were associated with notably increased sodium intakes on the days they were consumed. Current dietary guidelines in Ireland recommend limiting ‘processed meats such as bacon or ham’ due to their high salt content [37]. Adults who consumed large portions of restaurant-prepared ethnic dishes showed the highest sodium intakes for any of the foods examined on the days the dishes were consumed (4334 mg/10 MJ compared with a mean value of 3191 mg/10 MJ for the whole group), which likely reflects the use of salt and sodium-based flavour enhancers (e.g., monosodium glutamate) in the preparation of these dishes.

Larger portions of RTEBCs were associated with increased intakes of iron, folate and other B-vitamins for which results are not published (thiamin, riboflavin, niacin and vitamin B_6_) on the days they were consumed. The addition of micronutrients to RTEBCs has previously been credited with significantly contributing to micronutrient intakes in adults in Ireland [40] and elsewhere [41,42]. In the current work, the mean total folate intake associated with the consumption of large portions of RTEBCs was the highest for any of the foods examined (700 µg/10 MJ). This equates to an actual folate intake of 521 µg/day, exceeding the recommended dietary intake of 400 µg/day for both men and women [12]. This is of interest, inadequate intakes of dietary folate have been reported in Irish women, and less than 2% of Irish women aged 18 to 50 years consume the supplemental folic acid (400 µg/day) recommended to reduce the risk of neural tube defects in infants [12]. 

Larger portions of sugar-sweetened beverages were associated with decreased intakes of calcium, iron, folate and vitamin D, as well as other micronutrients for which results are not published (zinc, thiamin, riboflavin, niacin and vitamins A, C, E and B_6_). Data from the American National Health And Nutrition Examination Study (NHANES) have previously shown that increased consumption of ‘energy-dense, nutrient-poor foods’ (including sugar-sweetened beverages) results in marginal micronutrient intakes in adults, with such foods possibly consumed at the expense of more nutrient-dense foods [43]. In the current study, DED was higher than average on the days sugar-sweetened beverages were consumed, being particularly high amongst those consuming larger portions. Since DED was calculated to exclude all beverages, this suggests that while micronutrient intakes decrease with increasing portion size of sugar-sweetened beverages, it is not the case that less food is eaten on the days these beverages are consumed. Rather, it would appear that energy-dense, micronutrient-poor foods are being consumed along with the beverages. A striking inverse relationship was evident between the consumption of beer/cider and micronutrient intakes, particularly with increasing portion size of the beverages. Larger portions of beer/cider were associated with increased intakes of folate (which occurs naturally in the yeast used to brew beer), but with significantly decreased intakes of calcium, iron and vitamin D, plus other micronutrients for which results are not published (zinc, thiamin and vitamins A, C, E and B_12_). This is likely to reflect the dilution effect that occurs when energy-adjusted (rather than absolute) micronutrient intake values are presented, due to the additional energy provided by the alcohol. As such, the results do not suggest that foods are displaced when larger portions of beer/cider are consumed. In fact, DED values were higher than average on the days beer/cider was consumed—despite DED being calculated to exclude beverages—suggesting that beer/cider was consumed with other energy-dense foods.

Energy-dense, nutrient-poor foods are consistently associated with higher DED in adults in the literature [14,44,45,46]. The current work aligns closely these findings, showing larger portion sizes of biscuits, chocolate and sugar-sweetened beverages to be associated with higher DED.

There appear to be several distinct trends at play regarding the observations described in the present study. Firstly, larger food portion sizes may be associated with increased intake of a nutrient as a consequence of the composition of the food itself (e.g., larger portions of high-salt frying meats were associated with significantly increased Na intakes on the days they were consumed). The findings may also occur as a result of ‘food associations’, or from typical patterns of consumption. This might explain, for example, why DED was significantly higher on the days larger portions of sugar-sweetened beverages were consumed, although beverages themselves were excluded from the calculation of DED; it may be that larger portions of such beverages are typically consumed with other energy-dense foods, like fast food or confectionary products. Lastly, it is possible that some observations may be explained by food displacement. For instance, adults who consumed large portions of chocolate had decreased intakes of many micronutrients on the days it was consumed. As the chocolate cannot be responsible for the decreased micronutrient intakes per se, it is possible that the consumption of large portions of chocolate displaced other more micronutrient-dense snacks, such as fruit or yoghurt, from the diet on those days. 

The current analyses are based on food intake data that have been collected from a nationally representative sample of the population and quantified as accurately as possible. Although 43% of the food intake data were assigned weights using portion size estimation tools, they do represent, at present, the best available portion weight data for Irish adults. The work is novel for its focus on food portion size in relation to markers of dietary quality (rather than energy intake), and for examining a wide range of foods, rather than just energy-dense foods, which are more typically involved in discussions of food portion size. A possible limitation of this work is that it did not take into account the number of eating occasions per day for the foods examined. A recent study using cross-sectional data to examine the relative contributions of portion size, energy density and number of eating occasions to increased daily energy intakes in adults in the US (1977–2006) found that changes in portion size and number of eating occasions had accounted for most of the change [47], and so inclusion of this information may have provided a more complete picture on the relationship between food portion size and markers of dietary quality. It would also be interesting to look at food portion sizes in relation to dietary quality indices or scores, rather than individual markers of dietary quality, as a focus of future research.

As with all dietary surveys, energy under-reporting amongst participants was an issue to consider in the current study. Attempts were made to limit under-reporting by maintaining high researcher–participant interaction (three visits over the recording period). Nonetheless, 30% of participants were found to have under-reported energy intake. Under-reporters were kept in the analyses for two reasons. Firstly, as the aim of the study was to describe eating patterns in a nationally representative sample of Irish adults, the exclusion of all under-reporters may have introduced a secondary ‘selection bias’ to the work [48]. Secondly, the use of energy-adjusted, rather than absolute, nutrient intakes should have helped minimize the effects of energy under-reporting somewhat. It is worth mentioning, however, that this in turn may have slightly increased the micronutrient densities of foods that were accurately reported.

Reduction in portion size, particularly for energy-dense foods, to reduce energy intake and to help tackle the obesity epidemic is increasingly addressed in healthy eating guidelines. The findings of the current study provide a useful evidence base to support the portion-size guidance currently available for Irish adults. The study also identifies potential effects on dietary quality (both positive and negative) of varying portion sizes of different energy-dense foods, as well as other foods. This may help to further develop guidance on food portion sizes, e.g., relating guidance to foods that may be appropriate to consume in larger quantities. Findings from this study will also be of use to policy-makers in devising strategies to target intakes of specific nutrients going forward, particularly when considered in conjunction with previously published data on the typical portion weights of foods consumed by this group [17].

## 5. Conclusions

The current study describes observations between food portion sizes and markers of dietary quality in Irish adults on the days the foods were consumed. DED was higher and micronutrient intakes lower on the days larger portions of sugar-sweetened beverages were consumed. Intakes of micronutrients (excluding folate) were much lower on the days larger portions of beer/cider were consumed. Findings from the current work provide an evidence base from which more specific dietary guidance relating to portion size may be developed, and may be useful where strategies are being devised to target intakes of specific nutrients in the future.

## Figures and Tables

**Table 1:** Baseline demographic and lifestyle data for study sample of adults aged 18–64 years (*n* = 1274), Republic of Ireland ^1^.

	Males				Females				Total Group
	18–35 Years	36–50 Years	51–64 Years	Total Males	18–35 Years	36–50 Years	51–64 Years	Total Females	
	(*n* = 276)	(*n* = 205)	(*n* = 153)	(*n* = 634)	(*n* = 255)	(*n* = 232)	(*n* = 153)	(*n* = 640)	(*n* = 1274)
BMI ^2^ (kg/m^2^)	25.8	28.5	29.7	27.5	24.8	26.7	28.8	26.4	27.0
Overweight/obese (BMI > 25 kg/m^2^) (%)	52.5	81.1	85.7	69.5	38.3	55.1	70.5	52.0	60.5
Educated to third level (%)	42.0	43.8	42.6	42.7	66.7	54.2	43.6	56.7	49.7
Current smoker (%)	23.3	26.7	14.1	22.2	29.8	20.1	15.2	22.8	22.5

^1^ Small numbers of missing values occur in each category. ^2^ BMI, body mass index, was measured by researchers.

**Table 2:** Number of eating occasions (*n*) and median portion weights (g) consumed by portion size tertiles for foods included in the current study.

	*n*	Tertile Medians ^1^ (g)		
		T1	T2	T3
White bread and rolls	*2404*	50	76	125
Brown bread and rolls	*2454*	48	76	114
Potatoes, boiled	*1320*	112	181	280
Pizza	*310*	117	218	412
Ready-to-eat breakfast cereals (RTEBCs)	*1832*	30	45	72
Fruit, excluding dried fruit	*2218*	80	130	188
Vegetables, excluding pulses	*3094*	32	67	116
Baked beans	*326*	62	120	205
Frying meats (bacon, sausages, pudding)	*895*	30	50	106
Fish (fillets, uncoated)	*291*	71	126	200
Whole milk	*304*	200	300	568
Reduced-fat milks	*225*	180	260	488
Cheese	*1608*	20	36	60
Butter and spreads >59% fat	*927*	6	12	24
Low-fat spreads <38% fat	*781*	8	12	24
Biscuits	*1272*	18	30	52
Chocolate confectionary	*1182*	18	35	56
Sugary sweets	*218*	8	28	54
Crisps	*563*	25	25	45
Sugar-sweetened beverages	*969*	200	330	500
Beer/cider	*614*	568	1704	3444
Ethnic dishes	*127*	136	267	451

^1^ Tertile medians describe the median portion weights of particular foods in each of the ‘small’ (T1), ‘medium’ (T2) and ‘large’ (T3) portion weight tertiles.

**Table 3:** Mean daily nutrient intakes by tertile of food portion size on the days the foods were consumed by a nationally representative sample of adults aged 18–64 years (*n* = 1274), Republic of Ireland ^1^.

Mean Value (Whole Group)	Saturated Fat			*p-Trend*	Dietary Fibre			*p-Trend*	DED ^3^			*p-Trend*	Na			*p-Trend*
	%TE ^2^				g/10 MJ				kJ/g				mg/10 MJ			
		12.9				23.4				7.43				3191		
	T1	T2	T3		T1	T2	T3		T1	T2	T3		T1	T2	T3	
White bread and rolls	13.5	13.7	13.2		21.2	19.8	19.5	↓	7.59	8.01	8.36	↑	3180	3290	3470	↑
Brown bread and rolls	12.8	12.7	12.5		25.3	26.3	28.8	↑	7.02	7.25	7.26		3290	3280	3390	↑
Potatoes, boiled	12.6	12.8	12.8		25.6	25.2	26.7		6.66	6.50	6.11	↓	3120	3070	2990	
Pizza	13.1	13.5	12.7		22.2	18.6	19.5		8.39	9.36	9.27		3460	3370	3350	
Ready-to-eat breakfast cereals	12.7	12.7	12.5		23.0	24.4	25.2	↑	7.58	7.51	8.10	↑	3160	3070	3270	
Fruit, excluding dried fruit	12.6	11.9	11.9	↓	25.2	28.4	30.5	↑	6.92	6.37	5.83	↓	3130	3140	3010	
Vegetables, excluding pulses	13.1	12.7	12.8		22.3	24.9	27.4	↑	7.71	6.92	6.23	↓	3190	3140	3120	
Baked beans	13.2	13.4	11.2	↓	25.6	30.4	38.1	↑	7.85	7.33	6.92	↓	3470	3830	4160	↑
Frying meats	13.3	13.8	15.0	↑	21.7	19.5	19.2	↓	7.63	8.26	8.59	↑	3550	3860	4280	↑
Fish (fillets, uncoated)	11.7	11.5	11.3		25.4	27.4	26.9		6.66	6.23	6.27		2800	2740	2890	
Whole milk	14.2	15.6	16.5	↑	19.9	19.2	18.1		7.89	7.52	7.75		3130	3000	3040	
Reduced-fat milks	12.9	11.6	14.5		25.5	22.2	20.9		6.94	7.22	7.63		3430	3340	3130	
Cheese	12.9	14.4	16.3	↑	24.0	22.5	20.8	↓	7.41	7.74	8.08	↑	3300	3350	3410	
Butter and spreads >59% fat	13.5	15.2	18.2	↑	23.2	21.8	20.7		7.30	7.70	8.21	↑	3070	3160	3190	
Low-fat spreads <38% fat	10.9	11.6	12.0		27.8	26.0	25.2		6.72	7.10	7.21		3280	3450	3510	
Biscuits	13.0	13.3	13.8		24.5	24.4	22.9		7.09	7.51	8.12	↑	3120	3140	3110	
Chocolate confectionary	13.3	14.1	15.1	↑	23.4	22.3	20.5	↓	7.51	7.97	8.42	↑	3080	3000	2790	↓
Sugary sweets	12.4	12.7	13.3		24.0	21.4	18.5	↓	7.15	7.68	8.33		3110	2970	2790	
Crisps	14.0	14.0	14.6		22.3	22.2	23.7		8.53	8.56	8.71		3280	3210	3220	
Sugar-sweetened beverages	12.9	13.1	11.8	↓	20.8	18.3	16.7	↓	7.97	8.63	8.53	↑	3110	2990	2850	
Beer/cider	11.9	10.6	8.6	↓	20.2	17.0	13.0	↓	7.79	7.82	8.10		2950	2870	2420	↓
Ethnic dishes	10.8	11.1	10.0		17.7	18.1	21.9		8.25	7.94	7.49		3160	3560	4330	↑
**Mean Value (Whole Group)**	**Ca**			***p-Trend***	**Fe**			***p-Trend***	**Folate**			***p-Trend***	**Vitamin D**			***p-Trend***
	**mg/10 MJ**				**mg/10 MJ**				**µg/10 MJ**				**µg/10 MJ**			
		**1130**				**17.6**				**528**				**5.4**		
	**T1**	**T2**	**T3**		**T1**	**T2**	**T3**		**T1**	**T2**	**T3**		**T1**	**T2**	**T3**	
White bread and rolls	1090	1120	1150	↑	16.4	17.7	14.8	↓	443	438	377	↓	5.0	4.7	3.9	↓
Brown bread and rolls	1110	1150	1190	↑	16.4	16.9	18.1	↑	461	497	527		5.6	5.9	5.3	
Potatoes, boiled	1110	1140	1100		16.1	18.1	17.5		465	473	543		5.6	6.6	5.6	
Pizza	1300	1340	1460		17.0	13.5	13.2		416	326	302	↓	5.7	2.7	2.2	
Ready-to-eat breakfast cereals	1170	1220	1210		20.7	21.8	23.0	↑	484	518	700	↑	5.3	5.4	6.2	
Fruit, excluding dried	1140	1190	1190		16.7	19.3	16.7		452	484	485		5.5	6.6	6.6	
Vegetables, excluding pulses	1140	1120	1130		16.8	17.5	17.5	↑	420	503	508	↑	5.3	5.3	6.6	
Baked beans	1070	1100	1090		20.6	21.4	16.2		438	426	468		7.1	4.6	5.9	
Frying meats	1040	1010	1010		17.3	14.7	16.5		433	391	378	↓	5.3	4.5	4.8	
Fish (fillets, uncoated)	1270	1120	1070	↓	16.9	17.4	14.6		482	506	473		11.1	17.3	14.4	
Whole milk	1150	1330	1520	↑	13.3	13.7	12.6		407	381	372		4.2	5.4	6.9	
Reduced-fat milks	1420	1570	1770	↑	15.8	21.6	13.6		561	533	547		6.1	7.4	7.0	
Cheese	1180	1290	1490	↑	17.0	15.6	15.8	↓	435	433	409		4.5	4.4	5.1	
Butter and spreads >59% fat	1090	1040	1070		17.5	16.3	14.7	↓	397	389	382	↓	4.4	5.1	4.5	
Low-fat spreads <38% fat	1200	1230	1200		18.2	17.3	23.0		528	556	630	↑	6.0	7.0	8.4	↑
Biscuits	1170	1160	1050	↓	17.6	20.0	15.5		493	474	405	↓	6.2	5.3	5.0	
Chocolate confectionary	1140	1130	1060		17.9	16.5	15.1	↓	458	403	488		5.1	4.5	4.4	↓
Sugary sweets	1130	1150	1000		16.1	13.4	24.7		471	379	347		7.8	4.3	4.8	
Crisps	1150	1040	930	↓	19.6	15.1	13.6		433	383	378		4.3	3.7	3.8	
Sugar-sweetened beverages	1000	962	889		17.9	17.6	14.6	↓	403	362	418	↓	4.5	3.0	3.0	↓
Beer/cider	967	942	766	↓	15.4	12.7	12.5	↓	422	470	479	↑	3.7	3.5	2.2	↓
Ethnic dishes	853	890	833		16.2	23.2	19.7		345	335	283		3.6	4.4	4.8	

^1^ All nutrient intakes energy-adjusted apart from DED;^ 2^ %TE, percentage of total energy; ^3^ DED, dietary energy density; T1, T2 and T3 describe mean values of the dietary quality indicator being examined for each of the small.(T1), medium (T2) and large (T3) portion size tertiles; Differences across tertiles calculated using analysis of variance (ANOVA) with a Tukey post-hoc test (normal data) or Kruskal–Wallis with a post hoc Mann–Whitney U Test (non-normal data); ‘↑’ denotes a significant increase in values with increasing portion size. ‘↓’ denotes a significant decrease in values with increasing portion size; Significance accepted at the level of *p* < 0.05.
